# Supplementary material for: Decoding the Reproductive System of the Olive Fruit Fly, Bactrocera oleae
Source: Genes (Basel). 2021 Feb 28;12(3):355. doi: 10.3390/genes12030355 (PMC7997189; doi:10.3390/genes12030355)
Supplement: Supplementary file 1 [file genes-12-00355-s001.zip › genes-1107252-supple/genes-1107252-supplementary/Supplementary file S6.docx]

| Sample name | Number of reads | % aligned reads |
| --- | --- | --- |
| FAGs_B1 | 14085863 | 71.22% |
| FAGs_B2 | 13821053 | 46.65% |
| MAGS_B1 | 17617646 | 53.19% |
| MAGS_B2 | 18047757 | 58.24% |
| FAGsA1 | 19345731 | 68.66% |
| FAGsA2 | 15034748 | 62.23% |
| MAGSA1 | 20140852 | 65.64% |
| MAGSA2 | 13984792 | 58.52% |
| Testes_A | 28582348 | 31.15% |
| Testes_B | 23595947 | 28.96% |

Table S6.1: The table shows the number of reads and the % of aligned reads between all samples used for cDNA libraries.

Figure S6.1: A Principal component analysis showing the distribution of the samples used for the RNAseq analysis.
